# Efficient quantitative assessment of facial paralysis using iris segmentation and active contour-based key points detection with hybrid classifier

**DOI:** 10.1186/s12880-016-0117-0

**Published:** 2016-03-12

**Authors:** Jocelyn Barbosa, Kyubum Lee, Sunwon Lee, Bilal Lodhi, Jae-Gu Cho, Woo-Keun Seo, Jaewoo Kang

**Affiliations:** 1 Department of Computer Science and Engineering, Korea University, Seoul, South Korea; 2 Department of Information Technology, Mindanao University of Science and Technology, Cagayan de Oro (on-study leave), Philippines; 3 Department of Neurology, College of Medicine, Korea University Guro Hospital, Seoul, South Korea; 4 Department of Otorhinolaryngology-Head and Neck Surgery, Korea University Guro Hospital, Seoul, South Korea

**Keywords:** Facial image analysis, Facial paralysis measurement, Iris segmentation, Key point detection, Localized active contour, Hybrid classifier

## Abstract

**Background:**

Facial palsy or paralysis (FP) is a symptom that loses voluntary muscles movement in one side of the human face, which could be very devastating in the part of the patients. Traditional methods are solely dependent to clinician’s judgment and therefore time consuming and subjective in nature. Hence, a quantitative assessment system becomes apparently invaluable for physicians to begin the rehabilitation process; and to produce a reliable and robust method is challenging and still underway.

**Methods:**

We introduce a novel approach for a quantitative assessment of facial paralysis that tackles classification problem for FP type and degree of severity. Specifically, a novel method of quantitative assessment is presented: an algorithm that extracts the human iris and detects facial landmarks; and a hybrid approach combining the rule-based and machine learning algorithm to analyze and prognosticate facial paralysis using the captured images. A method combining the optimized Daugman’s algorithm and Localized Active Contour (LAC) model is proposed to efficiently extract the iris and facial landmark or key points. To improve the performance of LAC, appropriate parameters of initial evolving curve for facial features’ segmentation are automatically selected. The symmetry score is measured by the ratio between features extracted from the two sides of the face. Hybrid classifiers (i.e. rule-based with regularized logistic regression) were employed for discriminating healthy and unhealthy subjects, FP type classification, and for facial paralysis grading based on House-Brackmann (H-B) scale.

**Results:**

Quantitative analysis was performed to evaluate the performance of the proposed approach. Experiments show that the proposed method demonstrates its efficiency.

**Conclusions:**

Facial movement feature extraction on facial images based on iris segmentation and LAC-based key point detection along with a hybrid classifier provides a more efficient way of addressing classification problem on facial palsy type and degree of severity. Combining iris segmentation and key point-based method has several merits that are essential for our real application. Aside from the facial key points, iris segmentation provides significant contribution as it describes the changes of the iris exposure while performing some facial expressions. It reveals the significant difference between the healthy side and the severe palsy side when raising eyebrows with both eyes directed upward, and can model the typical changes in the iris region.

## Background

**Fig. 1:**
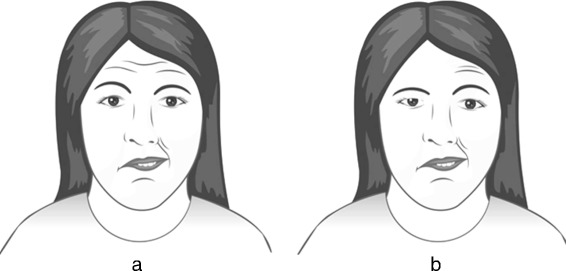
**a** Right-sided central palsy. **b** Right-sided peripheral palsy

Facial nerve palsy is a loss of the voluntary muscles movement in one side of the human face. It is frequently encountered in clinical practices which can be classified into two categories: peripheral and central facial palsy. Peripheral facial palsy is the result of a nerve dysfunction in the pons of the brainstem where the upper, middle and lower one side of facial muscles are affected while central facial palsy is the result of nerve function disturbances in the cortical areas where the lower half of one side of the face is affected but the forehead and eyes are spared, unlike in peripheral FP (Fig. 1) [1, 2].

Facial paralysis (FP) afflicted individuals suffer from inability to mimic facial expressions. This symptom creates not only dysfunctions in facial expression but also some difficulties in communication. It often causes patients to be introverted and eventually suffer from social and psychological distress, which can be even more severe than the physical disability [3]. This scenario has led to greater interest to researchers and to clinicians in this field, and consequently, to the development of grading facial functions and methods in monitoring the effect of medical, rehabilitation or surgical treatment.

There has been considerable body of work developed to assess facial paralysis. Some of the latest and widely used subjective methods are Nottingham system [4], Toronto facial grading system (TFGS) [5, 6], 1inear measurement index (LMI) [7], House-Brackmann (H-B) [8] and Sunnybrook grading system [9]. However, traditional grading systems are highly dependent to clinician’s subjective observation and judgment; thus, suffer from inherent drawback of being prone to intra and inter-rater variability [4, 6, 10, 11]. Moreover, these methods have issues in integration, feasibility, accuracy and reliability and in general are not commonly employed in practice [9]. Hence, an objective grading system becomes apparently invaluable for physicians to begin the rehabilitation process. Such grading system can be very helpful in discriminating between peripheral and central facial palsy as well as predicting the degree of severity. Moreover, it may assist the physicians to effectively monitor the progress of the patient in subsequent sessions.

In response to the need for objective grading system, many computer-aided analysis systems have been created to measure dysfunction of one part of the face and the level of severity, but none of them tackles the facial paralysis type as the classification problem. Classifying each case of facial nerve palsy into central or peripheral plays a significant role rather than just assessing the degree of the FP. This is to assist the physicians to decide for the most appropriate treatment scheme to use. Furthermore, most of the image processing methods used are labor-intensive, if not; suffer from the sensitivity to the extrinsic facial asymmetry caused by orientation, illumination and shadows. Thus, to create a clinically usable and reliable method is challenging and still in progress [1].

We proposed a novel method that enables quantitative assessment of facial paralysis that tackles classification problem of facial paralysis type and degree of severity. Maximum static response assay (MSRA) [12] assesses facial function by measuring the displacement of standard reference points of the face. It compares facial photographs taken at rest and at maximum contraction. The method was labor-intensive and time-consuming [13]. Watchman et al. [14, 15] measured facial paralysis by examining the facial asymmetry on static images. Their approach is sensitive to the extrinsic facial asymmetry caused by orientation, illumination and shadows [16]. Wang et al. [17] used salient regions and eigen-based method to measure the asymmetry between the two sides of face and compare the expression variations between the abnormal and normal sides. SVM is employed to produce the degree of paralysis.

Anguraj, K. et al. [18] utilize canny edge detection technique to evaluate the level of facial palsy clinical symptoms (i.e. normal, mild or severe). Nevertheless, canny edge detection is very vulnerable to noise disturbances. Input facial images may contain noise disturbances such us wrinkles or excessive mustache that may also result to many false edges detected. On the other hand, Dong, J. et al. [19] utilize salient point detection and Susan edge detection algorithm as the basis for quantitative assessment of patient’s facial nerve palsy. They apply K-means clustering to determine 14 key points. However, this falls short when this technique is applied to elder patients, in which exact points can be difficult to find [20]. Most of these works are solely based on finding salient points on human’s face with the use of the standard edge detection tool (e.g. Canny, Sobel, SUSAN) for image segmentation.

Canny edge detection may result in inaccuracy of edge detection and influences a connected edge points since this algorithm compares the adjacent pixels on the gradient direction to determine if the current pixel has local maximum. This may in turn result to improper generation of key points. Another method [20] was proposed based on the comparison of multiple regions on human face, where they compare the two sides of the face and calculate four ratios, which is used to represent the paralysis degree. Nevertheless, this method suffers from the influence of uneven illumination. A technique that generate closed contours for separating outer boundaries of an object from background such as LAC model for feature extraction may reasonably reduce these drawbacks.

In this study, we make three main contributions. First, we present a novel approach for efficient quantitative assessment of facial paralysis classification and grading. Second, we provide an efficient way for detecting the landmark points of the human face through our improved LAC-based key point detection. Third, we study in depth the effect of combining the iris behavior and the facial key point-based symmetry features on facial paralysis classification. In our proposed system, we leverage the localization of active contour (LAC) model [21] to extract facial movement features. However, to improve the segmentation performance of LAC, we present a method that automatically selects appropriate parameters of initial evolving curve for each facial feature; thereby improving the key points detection. We also provide an optimized Daugman’s algorithm for efficient iris segmentation. To the best of our knowledge, our work is the first to address facial palsy classification and grading using the combination of iris segmentation and key-point detection.

## Methods

### Proposed facial paralysis assessment: an overview

**Fig. 2:**
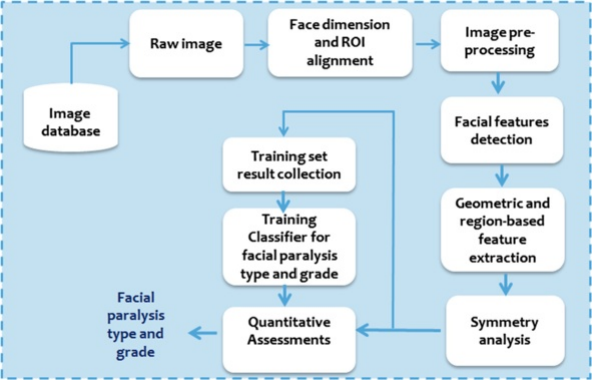
Framework of the proposed facial paralysis assessment

Our work evaluates facial paralysis by asymmetry identification in both sides of the human face. We capture facial images (i.e. still photos) of the patients with a front-view face and with reasonable illumination of lights so that each side of the face achieves roughly similar amount of lighting. The patient is requested to perform ‘at rest’ face position and four voluntary facial movements that include raising of eyebrows, closing of eyes gently, screwing-up of nose, and showing of teeth or smiling. The photo taking procedure starts with the patient at rest, followed by the four movements. A general overview of the proposed system is presented in Fig. 2. Facial images of a patient, which are taken while being requested to perform some facial expressions, are stored in the image database.

**Fig. 3:**
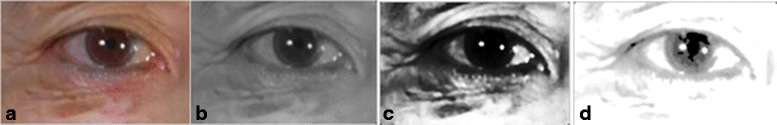
Pre-processing results. **a** original ROI, (**b**)–(**c**) median filter and histogram equalization results, respectively, (**d**) log transformation result with c = 0.1

The process starts by taking the raw image from the database. This is followed by face dimension alignment. At this step, we find the face region as our region of interest by performing face detection algorithm. As a result, we only keep the face region and all other parts of the captured images are removed. Preprocessing of images for contrast enhancement and noise removal is then performed. But firstly, the images or region of interest (ROI) have to be converted to a grayscale form. Median filtering technique and histogram equalization operation are then applied to remove noise and to obtain satisfactory contrast, respectively. Further image enhancement is achieved by applying the log transformation technique, which expands values of dark pixels and compresses values of bright pixels, essential for subsequent processes. Figure 3 shows an illustrative example of these pre-processing steps.

This is followed by facial features detection (e.g. eyes, nose, and mouth) and feature extraction. Features are extracted from the detected iris region and the key points. We then calculate the differences between two sides of the face. The symmetry of facial movement is measured by the ratio of the iris exposure as well as the vertical distances between key points from the two sides of the face. The ratios generated are stored in a feature set vector, which are trained by classifiers. Six classifiers (i.e. using rule-based and regularized logistic regression) were trained, one for healthy or unhealthy discrimination, one for facial palsy classification and another four classifiers for the facial grading based on House-Brackmann (H-B) scale.

### Feature extraction with optimized Daugman’s integro-differential operator and localized active contour

#### Face region detection

The facial images sometimes do not only include the face region only. Captured images may also include other parts such as the shoulder, neck, ears, hair or even background. Since we are only interested in the face region, it is our objective to keep this region and remove unnecessary parts of the captured images. To achieve this aim, we apply facial feature detection using Haar classifiers [22]. To detect human facial features, such as the mouth, eyes and nose, Haar classifier cascades are first to be trained. In order to train the classifier, AdaBoost algorithm and Haar feature algorithm were implemented. The Haar cascade classifier makes use of the integral and rotated images. Integral image [23] is an intermediate representation of an image and using this, the simple rectangular features of a certain image are calculated. Integral image is an array that contains the sums of the pixels’ intensity values located directly to the left of a pixel and directly above the pixel at location (x, y) inclusive. Thus, on the assumption that G[x, y] is the pre-specified image and GI[x, y] is the integral image then the formula for computing the integral image is as follows:

(1) $$  GI\left[ {x,y} \right] = \sum \limits_{{\mathrm{x'}} \le {\mathrm{x}},{\mathrm{y'}} \le y} G\left({\mathrm{x'},{\mathrm{y'}}} \right)  $$

The integral image is rotated and is calculated at a forty five degree angle to the left and above for the x value and below for the y value. If GR[x, y] is the rotated integral image then the formula for computing the rotated integral image is as follows:

(2) $$  GR\left[ {x,y} \right] = \sum \limits_{{\mathrm{x'}} \le {\mathrm{x}},{\mathrm{x'}} \le {\mathrm{x}} - \left| {{\mathrm{y}} - {\mathrm{y'}}} \right|}^{} G\left({\mathrm{x'},{\mathrm{y'}}} \right)  $$

Using the appropriate integral image and taking the difference between six to eight array elements forming two or three connected rectangles, a feature of any scale can be computed. This technique can be adapted to accurately detect facial features. However, the area of the image that is subject for analysis has to be regionalized to the location with the highest probability of having the feature. To regionalize the detection area, regularization [22] is applied. By regionalizing the detection area, false positives are eliminated and the detection latency is decreased due to the reduction of the region examined.

#### Feature extraction process

**Fig. 4:**
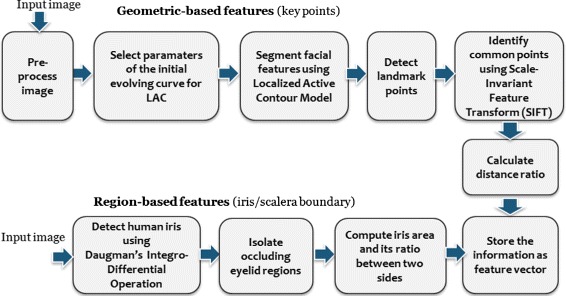
Flow of feature extraction process

Once the facial regions are detected, feature extraction process takes place. This process involves detections of key points and iris/sclera boundaries. Figure 4 shows the flow on how features are extracted.

Feature extraction start from the preprocessing of the input image and facial region detection. To extract the geometric-based features, parameters of the initial evolving curve of each facial feature (e.g. eyes, eyebrows and lip) are first automatically selected. These parameters are then used as inputs to localized active contour model [21] for proper segmentation of each facial feature. This step is followed by the landmarks or key point detection process. We also apply Scale-invariant feature transform (SIFT) [24] to find the common interesting point of two images (i.e. at rest position and eye brows lifting). The points generated by SIFT are useful for determining the capability of the patients to do facial motions by comparing the two facial images that includes at rest position and lifting of eyebrows. Region-based features extraction involves detection of iris/sclera boundary using Daugman’s Integro-Differential Operation [25]. All features are stored in a feature vector. Table 1 shows the list of asymmetrical features we used in this paper. Labeled parts of facial features are shown in the subsection that tackles the key points detection.

**Table 1:** List of features

Asymmetrical features	Notation	Parameters
1) Iris area while lifting eyebrows with both eyes		
directed upward.	EBlift_Iris	f1
2) Rate of movement from at rest to lifting of eyebrows		
(using distance between SO and upper part of the occluded iris).	EBmd_SO_uIris	f2
3) Rate of movement from at rest to lifting of		
eyebrows (using distance between SO and IO).	EBmd_SO_IO	f3
4) Distance between SO and IO while lifting eyebrows.	EBlift_SO_IO	f4
5) Distance between SO and upper boundary of the		
occluded iris while raising eyebrows with both eyes looking upward.	EBlift_SO_uIris	f5
6) Distance between SO and IO while closing both eyes.	Eclose_SO_IO	f6
7) Iris area while showing teeth or smiling.	smile_Iris	f7
8) Distance between IO and mouth angle		
while smiling.	smile_IO_MA	f8
9) Iris area while screwing nose.	snarl_Iris	f9
10) Mean ratio of features 1–9.	meanRatio	f10

#### Key points detection

**Fig. 5:**
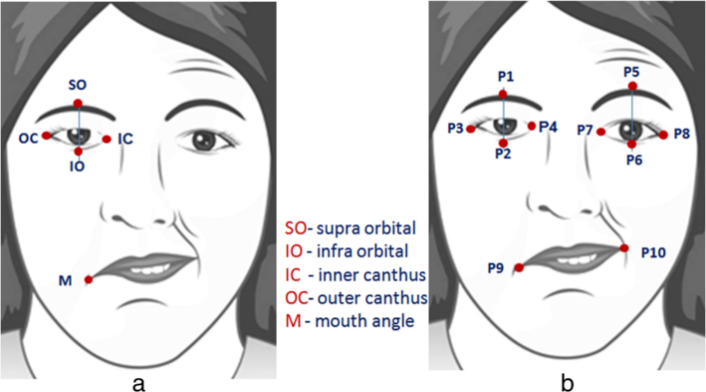
**a** Labeled parts of facial features, (**b**) key points

The detection of key points includes initialization and contour extraction phases for each facial feature we used in this paper. The goal is to find the 10 key points on edges of facial features as shown in Fig. 5a and b.

##### Overview of localized region-based active contour model (LACM)

This section provides the overview of the primary framework of LAC [21] model, which establishes an assumption that the foreground and background regions would be locally different. The statistical analysis of local regions leads to the construction of a family of local energies in every point along the evolving curve. In order to optimize these local energies, each point is considered individually, and moves to minimize (or maximize) the energy computed in its own local region. To calculate these local energies, local neighborhoods are split into local interior and local exterior region by the evolving curve.

In this paper, we let *I* be a specified image on the domain *Ω*, and *C* be a closed contour represented as the zero level set of a signed distance function *ϕ*, whose value can be given as: C ={*w*|*ϕ*(*w*)} [26]. The interior of *C* is specified by the following approximation of the smoothed Heaviside function:

(3) $$ H\phi (w) = \left\{ {\begin{array}{lc} \quad\;\, 1,\qquad \qquad\qquad\quad\quad\; \phi (w) < - \varepsilon\\ \quad\;\;\; 0,\qquad\qquad\qquad\quad\quad\; \phi (w) < \varepsilon\\ {\frac{1}{2}\left\{ {1 + \frac{\phi }{\varepsilon} + \frac{1}{\pi }\sin \left({\frac{{\pi \phi (w)}}{\varepsilon }} \right)} \right\},otherwise.} \end{array}} \right.  $$

Similarly, the exterior *C* can be defined as 1- *Hϕ*(*w*). The epsilon *ε* is the parameter in the definition of smooth Dirac function having a default value of 1.5. The area just adjacent to the curve is specified by finding the derivative of *Hϕ*(*w*), a smooth version of the Dirac delta denoted as

(4) $$ \delta \phi (w) = \left\{ {\begin{array}{lc} \quad\;\; 1,\qquad\qquad\qquad {\phi (w) = \varepsilon }\\ \quad\; 0,\qquad\qquad\qquad {\left| {\phi (w)} \right| < \varepsilon }\\ {\frac{1}{{2\varepsilon }}\left\{ {1 + \cos \left({\frac{{\pi \phi (w)}}{\varepsilon}} \right)} \right\},otherwise.} \end{array}} \right.  $$

Parameters *w* and *x* are expressed as independent spatial variables. Each of these parameters represents a single pointing, respectively. Using this notation, the characteristic function *β*(*w*,*x*) in terms of a radius parameter *r* can be written as follows:

(5) $$ \beta (w,x) = \left\{\begin{array}{cc} {1,}& \quad {\left\| {w - x} \right\| < r}\\ {0,}& \quad {otherwise.} \end{array} \right.  $$

*β*(*w*,*x*) is then utilized to mask local regions. Therefore, a localized region-based energy formed from the global energy by substituting local means for global ones is shown below [27]:

(6) $$ F = - {\left({ u_{w} - v_{w}} \right)^{2}},  $$

(7) $$ u_{w} = \frac{{\int_{\Omega_{x}} {\beta (w,x) \cdot (H\phi (x)) \cdot I(x)dx} }}{{\int_{\Omega_{x}} {\beta (w,x) \cdot (H\phi (x))dx} }}  $$

(8) $$ v_{w} = \frac{{\int_{\Omega_{x}} {\beta (w,x) \cdot (1 - H\phi (x)) \cdot I(x)dx} }}{{\int_{\Omega_{x}} {\beta (w,x) \cdot (1 - H\phi (x))dx} }}  $$

where the localized versions of the means *u*_*w*_ and *v*_*w*_ represent the intensity mean in the interior and exterior regions of the contour, which is localized by *β*(*w*,*x*) at point *x*. By ignoring the image irregularity that may arise outside the local region, we only consider the contributions from the points within the radius *r*. Also, a regularization term is added to maintain the smoothness of the curve. Additionally, the arc length of the curve is penalized and weighted by a parameter *λ* and the final energy E (*ϕ*) is given as follows:

(9) $$ \begin{aligned} E(\phi) &= \int_{\Omega_{w}} {\delta \phi (w)} \int_{\Omega_{w}} {\beta (w,x) \cdot F(I(x),\phi (x))dxdw}\\&\quad + {\lambda} \int_{\Omega_{w}} {\delta \phi (w)} \left\| {\nabla (w)} \right\|dw \end{aligned}  $$

By taking the first variation of this energy with respect to *ϕ*, the following evolution equation is obtained:

(10) $$ \begin{aligned} \frac{{\partial \phi }}{{\partial t}}(w) &= \delta \phi (w)\int_{\Omega_{x}} {\beta (w,x) \cdot \nabla_{\phi (x)}} F(I(x),\phi (x))dx\\&\quad + \lambda \delta \phi (w)div\left({\frac{{\nabla \phi (w)}}{{\left| {\nabla \phi (w)} \right|}}} \right)\left\| {\nabla \phi (w)} \right\|. \end{aligned}  $$

It is worth note taking that this ensures that nearly all region-based segmentation energy can be put into this framework.

##### Initialization

In localized active contour approach, analysis of the local regions paves the way for the construction of local energies at each point along the curve. For the optimization of these local energies each point is considered separately, and moves to minimize the energy computed in its own local neighborhoods into local interior and local exterior by the evolving curve. This approach generally gives a satisfactory result in segmenting objects. However, such localization has an inherent trade-off because of its greater sensitivity to initialization [21]. Proper parameters (e.g. enough amount of the radius, distance from the evolving curve, etc.) have to be determined before fitting it in the localized active contour (LAC) model for correct segmentation.

**Fig. 6:**
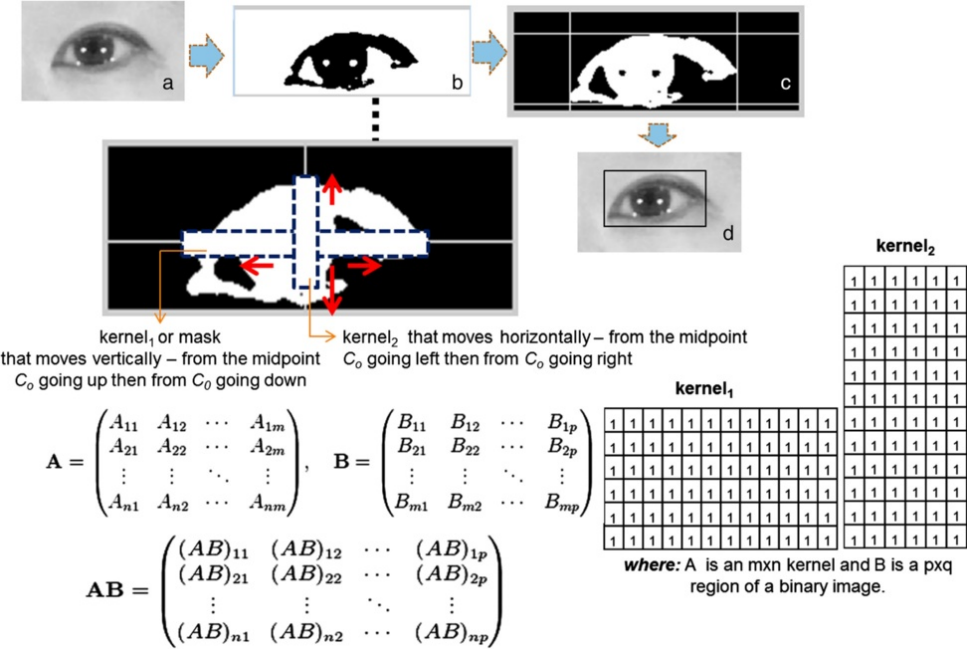
The procedure of finding the minimum-bounding rectangle: (**a**) eye image, (**b**) result of our algorithm, (**c**)–(**d**) minimum-bounding rectangular form

For finding the minimum-bound rectangular form of the eyes and eyebrows with proper parameters, we develop our own approach and the steps are summarized as follows: We choose the region of interest (ROI) based on the detected facial feature by Haar algorithm [22, 23]. For each ROI, we apply pre-processing for image improvement, which is to suppress unwanted distortions or enhances some image features essential for subsequent processes. This can be achieved by applying median filter, histogram equalization and log transformation techniques. To find the threshold that maximizes the between-class variance and transform the graylevel to a binary one, we choose OTSU’s algorithm [28]. To further remove the noise, we applied the 8-neighborhood rule implementation, which removes all connected components that have fewer than P pixels (e.g. 1 % of the area of an image used for cluster, i.e. image size * 0.01) from the binary image. From this result, we define a window kernel *m* x *n* in two forms: vertical and horizontal form. A window kernel, in this context, is a binary block of *m* x *n* size where *m* and *n* are the number of rows and columns respectively (i.e. all having a pixel value of 1). For example, to find the x-axis boundary of the initial evolving curve *c*, *k* is run from center point going to the left, then to the right until convergence. In each step, we calculate the sum of the product of each pixel of *k* and the pixel of the binary image. The algorithm has converged when the computed sum yields 0 (i.e. with a background set to 0 - black), which means that the kernel hits a certain blocks of an image. Similarly, to find the y-axis boundary of *c*, we run the same kernel *k* up then down and stop until convergence. The intuition is that the operation stops when the kernel finds a matching shape. Sample output is shown Fig. 6. The size of the kernel depends on the result after 8-neighborhood rule implementation. For automatic selection of parameters for initial evolving curve of lip facial feature, we apply the algorithm in [29].

##### Segmentation using Localized Active Contour Model (LACM) and feature point location

When the minimum- bounding rectangle of each region is successfully identified, this rectangle shape is considered as the evolving curve that represents the zero level set C, as described in key point detection subsection, which can be fitted well in the LACM. Then the local neighborhoods of the points can be subsequently split by the evolving curve into two local regions: the local interior and local exterior region.

The basic idea is to allow a contour to deform in order to minimize a given energy functional so that the desired contour extraction is achieved. By computing the local energies at each point along the curve, the evolving curve will deform by minimizing the local energies. The steps of facial feature contour extraction are as follows: Locate the eyes, eyebrows and lip region;

- Preprocess;
- Obtain the minimum-bounding rectangular form;
- Evolve with iteration;
- Extract the eyes, eyebrows and lip contours.

**Fig. 7:**
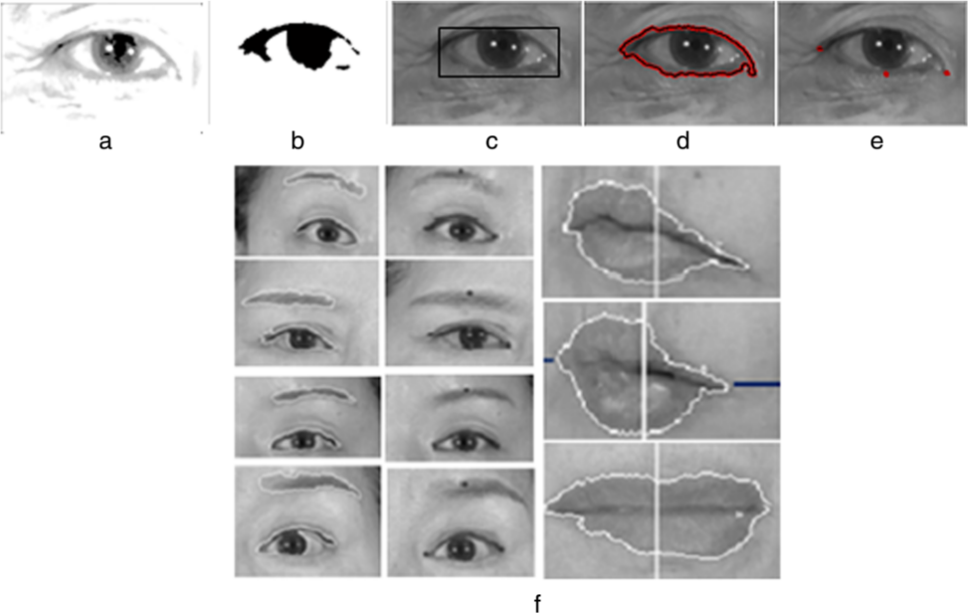
The procedure for facial feature contour extraction and key points detection: (**a**) pre-processing result, (**b**)–(**c**) results of our method for finding the minimum-bounding rectangular form, (**d**) the segmented object after 100 iterations with parameter *λ* at 0.3, (**e**) detected key-points, (**f**) more sample outputs of key points generated from the segmented region performed by a Localized Active Contour Model (LACM)

We are interested of the following key points: two corners of the mouth, the supra orbital (SO), infra orbital (IO), inner canthus (IC) and outer canthus (OC). In this paper, we identify these key points from the segmented facial features generated by our improved LAC model. We take the segmented object (i.e. in binary form) and utilize the idea of the distance transform technique. With this technique, each pixel of the binary image is assigned a number that is the distance between that pixel and the nearest nonzero pixel of the binary image. For example, to get the left corner of the mouth, we calculate the distance transform of the first half of the binary image; and the first coordinates that has an assigned value equal to 0 would be the left corner. Figure 7 shows the experimental samples of the facial features contour extraction and the key points generated by the proposed approach.

#### Iris detection

**Fig. 8:**
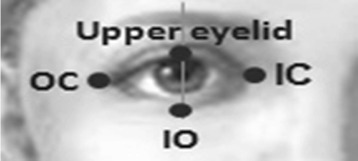
Eye edges and corners

A person having paralysis in one side of his face would likely to have asymmetric distance between the upper and lower eyelid while performing facial movements. Intuitively, they may also have uneven iris exposure when performing different such voluntary movements (e.g. screwing of nose, showing of teeth or smiling). We apply Daugman’s algorithm [25] and LACM to detect the iris boundary. From the detected face region, we need to determine the parameters of the eye region as input to Daugman’s algorithm. As such, we utilize the detected 4 key points that includes the upper eyelid, IO, IC, and OC as shown in Fig. 8.

##### Daugman’s algorithm

Daugman’s algorithm is by far the most cited method in the iris recognition literature. It was proposed in 1993 and was implemented effectively in a working biometric system [30]. In this method, the author assumes that both pupil and iris have circular form and the integro-differential operator. After automatic parameter selection (i.e. rectangular boundary) of each eye, we implemented two pre-process operations for image contrast enhancement purposes. First, we use histogram equalization to improve the contrast between each eye’s regions, which potentially facilitate the segmentation task. Second, we apply binarization based on a threshold, which is commonly used to maximize the separability of the iris regions from the rest of the eye region. However, this process has one major drawback of being highly dependent of the chosen threshold, and as image characteristics change, the results may seriously deteriorate [30]. Moreover, the binarization that compromises one of the Daugman’s algorithm is based on applying an integro-differential operator to find the iris and pupil contour. We find the equation below,

(11) $$ max\left({r,{x_{o}},{y_{o}}} \right)\left| {{G_{\sigma} }\left(r \right)*{\partial \over {\partial r}} \oint \limits_{r,{x_{o}},{y_{o}}}^{} {{I\left({x,y} \right)} \over {2\pi r}}ds} \right|  $$

where *X*_0_, *Y*_0_, r: the centre and radius of the circle (for each of pupil and iris); G *σ*(*r*): Gaussian function; *δ*r: the radius range; I(X, Y): the original iris image. G *σ*(*r*) is a smoothing function, the smoothed image is then scanned for a circle that has a maximum gradient change, which indicates an edge. The above algorithm is done twice, first is the iris contour extraction then the pupil contour extraction. It is worth note taking that the problem is that the illumination inside the pupil is a perfect circle with very high intensity level, i.e. almost pure white. Therefore, the problem of sticking to the illumination as the maximum gradient circle has to be addressed.

##### Optimized Daugman’s algorithm

To alleviate this problem, modification to the integro-differential operator is necessary to ignore all circles if any pixel on this circle has a value higher than a certain threshold. We apply the method proposed by P. Verma [30], where this threshold is determined to be 200 for the grayscale image. This is to ensure that only the bright spots, i.e. values usually higher than 245 will be rejected. On the other hand, iris is normally occluded by eyelid and eyelashes. For eyelid and eyelashes detection, P. Verma et al. [30] applied Sobel edge detection to the search regions to detect the eyelids. The eyelids are detected using linear Hough Transform method. The total number of edge points in every horizontal row inside the search region is calculated. In this paper, since we are only interested with the iris/sclera boundary, we simply apply the LAC model with our automatic parameter selection of the initial evolving curve to segment the eyes and get the portion of the eyelids. We then get the intersection of it with the detected iris/sclera boundary using Daugman’s algorithm [25]. In what follows; we describe our approach for detecting the iris:

1. Detect iris/sclera boundary. (Figures 9a and b)

**Fig. 9:**
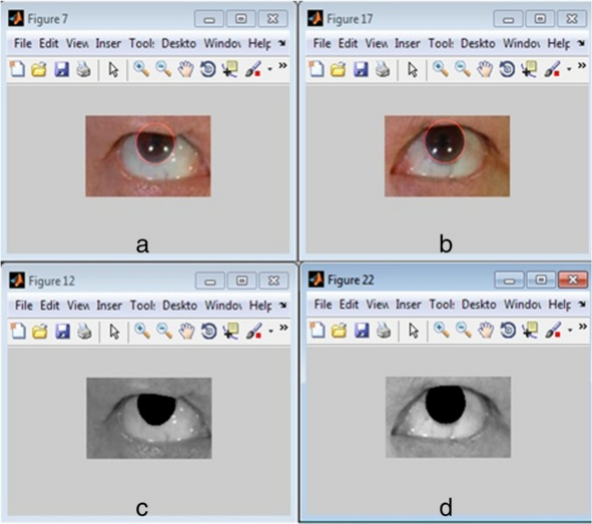
Extracted iris using optimized Daugman’s algorithm: (**a**)–(**b**) result of Daugman’s Integro-Differential operator iris detection. **c**–**d** result of optimized Daugman’s algorithm
2. Do binarization. We let this as vector A.
3. Take a fraction of the radius of the detected iris to create a rectangular bound making it as the initial evolving contour and fit it to LAC model.
4. Segment iris using LAC model.
5. Do binarization of the result of the active contour segmentation. We call it vector B.
6. Find intersections of two vectors A and B to get the values that are common to both vectors A and B. In set theoretic terms, we represent this as A B.This will return the values common to both A and B. This was followed by applying morphological operations: erosion followed by dilation.

**Fig. 10:**
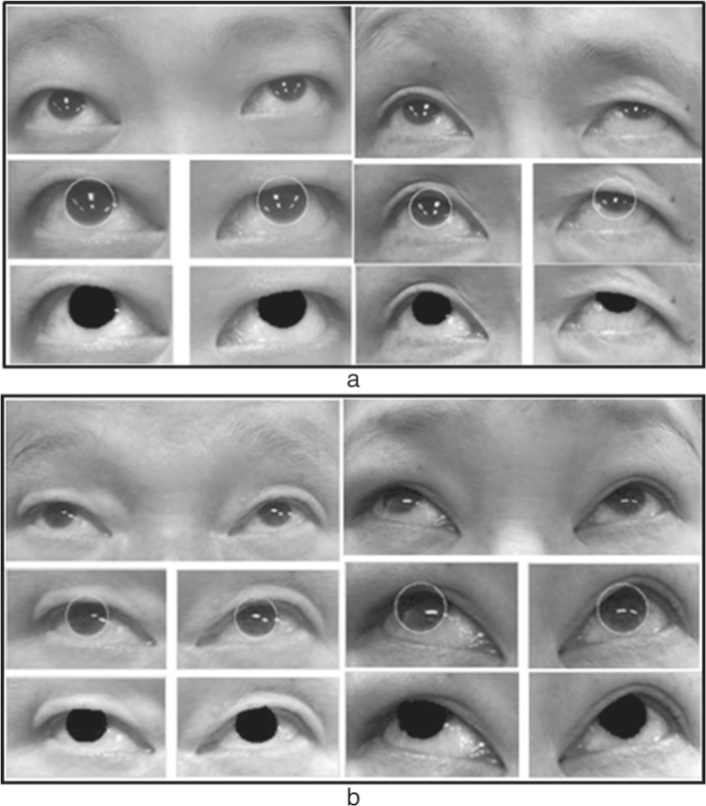
More sample results of optimized Daugman’s algorithm. **a** Peripheral palsy subjects (**b**) central palsy subjects

Take note that in iris segmentation in step 4, we tune the parameters: *λ*=0.1, *r*=12, and *iteration*=100; where *λ* is the relative weighting of curve smoothness, usually between [0 1], *r* is the radius of the ball used for localization and iterations is the number of iterations to run. Figures 9 and 10 depict the experimental samples of the iris segmentation by our optimized Daugman’s algorithm.

### Facial paralysis classification and grading

#### Symmetry measurement by iris and key points

In this study, we measure the symmetry of both sides of the face using the ratios we obtained from extracting iris exposure and the vertical distances between the key points on each side and store them in a feature vector. We capture a set of five facial images of each patient performing ‘at rest’ position and some voluntary movements that includes, raising of eyebrows (while looking upward), closing of eyes, screwing of nose and showing of teeth or smiling. Then we calculate the area of the extracted iris as well as the distances between the key points: *P*_1_*P*_2_,*P*_5_*P*_6_,*P*_2_*P*_9_ and *P*_6_*P*_1_0 (see Fig. 5b) and calculate the ratio between two sides. We find the expression below:

- irisA= *A*_*l*_/*A*_*r*_ if *A*_*l*_<*A*_*r*_;
- irisA = *A*_*r*_/*A*_*l*_ if *A*_*r*_<*A*_*l*_;
- dRatio = *Dist*_*l*_/*Dist*_*r*_ if *Dist*_*l*_<*Dist*_*r*_;
- dRatio = *Dist*_*r*_/*Dist*_*l*_ if *Dist*_*r*_<*Dist*_*l*_.

where *A*_*l*_ and *A*_*r*_ are the computed area or amount of iris exposure at the left and right side respectively; *Dist*_*l*_ and *Dist*_*r*_ are the computed distance of the specified points of each half of the face. irisA and dRatio are the ratio of the iris area and vertical distances respectively.

**Fig. 11:**
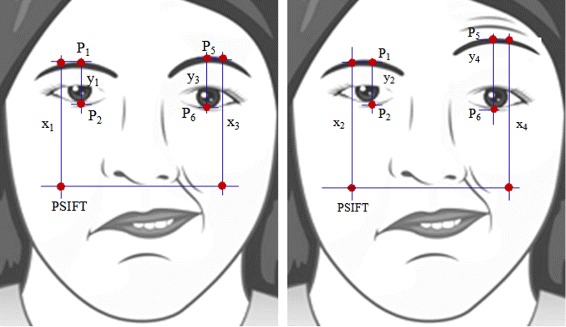
Symmetry measurement based on one of the common points (*PSIFT*) generated by Scale-invariant feature transform (SIFT)

Another important feature for symmetry measurement is the capability of the patients to raise the eyebrows (i.e. rate of movement feature in Table 1), by comparing the two facial images, the ‘at rest position’ and ‘raising of eyebrows’ as shown in Fig. 11. First, we pick one of the common points generated by SIFT algorithm, which are located below the eyes. We denote it as *PSIFT*. Then, we compute the vertical distances *x*_1_ and *y*_1_ (Fig. 11a), where *x*_1_ and *y*_1_ are the distances from *P*_1_ to *PSIFT* and *P*_1_ to *P*_2_ of the right eye, respectively. We then compute the ratio of *x*_1_ and *y*_1_. Similarly, for the second image (Fig. 11b), we calculate *x*_2_ and *y*_2_ as well as the ratio. We get the difference of these two ratios (i.e. difference between *y*_1_/ *x*_1_ and *y*_2_/ *x*_2_) and denote it as *rMovement*. The same procedure is applied to the two images for finding the ratio difference for the left eye (i.e. difference between *y*_3_/ *x*_3_ and *y*_4_/ *x*_4_) and denote it as *lMovement*. Intuitively, the difference of these two ratios for FP patients would likely to have a smaller value (usually approaching to 0, which implies inability to perform) than those of the normal subjects. Thus, the rate of movement can be computed by finding the ratio between *rMovement* and *lMovement*. The higher the value of this ratio, the higher possibility that the patient is able to raise his eyebrows, thereby signifying the ability to perform such activity.

#### Facial palsy classification

Classification of facial paralysis type involves two tasks: discriminating normal from abnormal subjects and the proper facial palsy classification. In this context, we need two classifiers to be trained, one for healthy and unhealthy discrimination (0-healthy, 1-unhealthy) and another one for facial palsy type classification, i.e. 0-peripheral palsy(PP), 1-central palsy(CP). For each classifier, we consider Regularized Logistic Regression (RLR), Support Vector Machine, Decision Tree (DT), and naïve bayes (NB) as appropriate classification methods as they have been used successfully for pattern recognition and classification on datasets with realistic size. In addition to these classification methods, we also consider a hybrid classifier (i.e. rule-based + RLR) as appropriate for carrying out the classification task.

We model the mapping of symmetry features (i.e. f1, f2, …, f10 as described earlier) into each of these tasks as a binomial classification problem. Although Machine learning (ML) approach has been proven to yield quite accurate classification results, our objective is to first find a classifier with high precision given a training data size that is not very large. Also, given the intuition that normal subjects would likely to have an average measurement ratio closer to 1.0 and central palsy patients would likely to have a distance from SO to IO and iris exposure ratio nearly close to 1, applying rule-based approach prior to employing ML method would be appropriate in our work. Hence, a hybrid classifier that combines a rule-based expert system and machine learning was applied to both tasks.

**Fig. 12:**
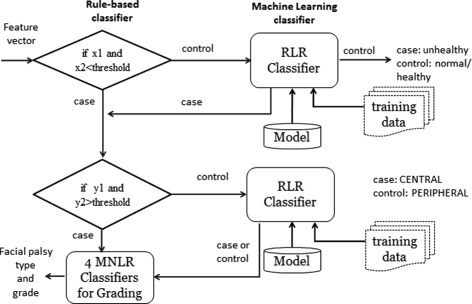
Hybrid classifier

This process is presented in Fig. 12. If rule number 1 is satisfied, the algorithm continues to move to the case path (i.e. for the second task), making a test if rule number 2 is also satisfied; otherwise, it performs a machine learning task, such as RLR, SVM, and NB. It is worth note taking that the rules are generated after fitting the training set to the DT model. For example, rule 1 may have conditions, like if f10 < 0.95 and f8 < 0.95 (where f10 and f8 are two of the predictors used - see Table 1), then the subject is most likely to be diagnosed with facial paralysis, and therefore can proceed for rule no. 2 (i.e. to predict the FP type); otherwise, it performs a machine learning task. If the classifier returns 0, the algorithm simply exits from the whole process (i.e. control as depicted in the figure) as this implies that the subject is classified as normal/healthy; otherwise, it goes to the case path (i.e. the 2nd task - facial palsy classification) for performing test for rule number 2.

If rule number 2 is met, the system returns 1 (i.e. 0-PP; 1-CP), otherwise the feature set is fed to another classifier, which could return either 0 or 1. Similar to rule number 1, rule number 2 is also generated by DT model. For example, rule 2 may have conditions like, if f1 > 0.95 and f4 > 0.95, then it is most like to be diagnosed as having central palsy (CP), otherwise, the feature set is fed to another classifier, which could return either 0 or 1 (i.e. 0-PP; 1-CP). For whichever type of facial paralysis the system returns, the algorithm continues to feed the features to assess the degree of paralysis in each region (detailed explanation is given in the next subsection).

#### Quantitative assessment

To assess the degree of paralysis in every region, we utilize the regional H-B scale that starts from grade I (normal) to grade VI (total paralysis). We model the mapping of the predictors into a regional H-B grade as a multi classification problem. Three hybrid classifiers are trained for the region grading: one classifier for the mouth region, one for the forehead region, and another one of the eye region. Finally, another hybrid classifier is employed for the overall FP grading. For region grading, we have the following features: forehead region utilizes f1, f2, f3, f4 and f5, mouth uses features f7, f8 and f9 and finally the eye region use f1 and f6. The next section presents the detailed steps for rule extraction, which is necessary in formulating our hybrid model. This enables us to get the grade of each region. Finally, to determine the degree of severity or overall grade, we utilize the H-B score [8]. We test each of the region grades (e.g. if mouthGrade =2, foreheadGrade =2 and eyeGrade =2, using to H-B scale [8], the overall grade is 3 - moderate). If the conditions are not satisfied the feature set is fitted to a multinomial logistic regression (MNLR) to get the overall grade.

## Results and discussion

In our experiments, 325 facial images were taken from 65 subjects that include 50 patients and 15 healthy subjects. 50 patients consist of 29 males and 21 females, whereas, healthy subjects contains 5 males and 10 females. Subjects come from different races that include Koreans and Filipinos, with age ranges from 19 to 82. From the 50 unhealthy subjects, 40 of which have peripheral palsy (PP) cases and 10 have central palsy (CP) cases. We used 70 % of the dataset as the training set and 30 % as the test set. For example, in discriminating healthy from unhealthy subjects, we used 45 subjects (i.e. 35 patients plus 10 normal subjects) as the training set and 20 subjects (i.e. 15 patients plus 5 normal) as the test set. While in FP type classification problem 35 unhealthy cases (i.e. 28 PP and 7 CP) as our training set and 15 (i.e. 12 PP and 3 CP) as our test set. Subjects with facial palsy symptoms like Bell’s palsy, left parotid tumor, Ramsay-Hunt syndrome were taken from Korea University, Guro Hospital. This study was approved by the Institution Review Board (IRB) of Korea University, Guro Hospital (with reference number MD14041-002). The board permitted not taking an informed consent due to the retrospective design of this study.

Each subject performs 5 facial movements in 2048 × 1152 resolutions, which are converted to 960 × 720 pixels during image processing. Their facial palsy type and the overall H-B grading were evaluated by the clinicians. We calculate the area of the extracted iris and the vertical distances between the key points: *P*_1_*P*_2_,*P*_5_*P*_6_,*P*_2_*P*_9_ and *P*_6_*P*_1_0. Overall, we utilize 10 features to train the classifier. Few sample results are presented in the Table 2. The samples are categorized into three parts: peripheral palsy (rows 1–4); central palsy (rows 5–8); and healthy (rows 9–12) cases. Notice that healthy subjects have very minimal asymmetry in both sides of the face yielding a ratio that approaches to 1.

**Table 2:** Some results after extracting features

	f1	f2	f3	f4	f5	f6	f7	f8	f9	f10
1	0.86	0.49	0.45	0.85	0.62	0.93	0.85	0.97	0.94	0.86
2	0.83	0.24	0.19	0.78	0.68	0.84	0.68	0.82	0.83	0.78
3	0.88	0.54	0.41	0.86	0.63	0.93	0.90	0.93	0.79	0.84
4	0.84	0.39	0.45	0.75	0.62	0.83	0.88	0.97	0.92	0.83
5	0.96	0.87	0.95	0.92	0.94	0.90	0.92	0.93	0.86	0.92
6	0.97	0.93	0.80	1.00	0.94	0.91	0.88	0.94	0.90	0.93
7	0.98	0.90	0.85	0.95	0.93	0.92	0.92	0.86	0.85	0.91
8	0.96	0.89	0.87	0.95	0.97	0.91	0.91	0.91	0.90	0.93
9	0.99	0.93	0.86	0.97	0.98	1.00	0.99	0.95	0.95	0.98
10	0.96	0.82	0.79	0.97	1.00	0.95	0.90	0.93	1.00	0.96
11	0.98	0.95	0.86	0.95	0.96	1.00	0.94	0.97	0.95	0.96
12	0.96	0.83	0.80	0.98	0.97	0.98	0.96	1.00	1.00	0.98

### Facial palsy classification and quantitative assessment of overall paralysis

SVM, regularized logistic regression (RLR), naïve bayes (NB), and classification tree (DT) were also utilized to compare with our hybrid classifiers. Since the size of the dataset was not huge, we adopt the k-fold cross-validation test scheme.The procedure involves 2 phases: rule extraction and hybrid model formation.

**Phase 1: rule extraction** Given the dataset D = (*x*_1_,*y*_1_,…,(*x*_*n*_,*y*_*n*_)), we hold out 30 % of D and use it as a test set T = ((*x*_1_,*y*_1_,…,(*x*_*t*_,*y*_*t*_)), leaving 70 % as our new dataset D’. We adopt k-fold cross-validation test scheme over the new dataset D’, i.e. with k = 9. For example, if N = 45 samples, each fold have 5 samples. In each fold, we leave one fold out as our validation set and use the remaining 8 folds as our training set (e.g. in the first round, fold 1 is the validation set, in the second round fold 2 is the validation set and so on and so forth). In each fold, we train the 8 folds to learn a model (e.g. extract rules). Since we have 9 folds, we do this procedure for 9 repetitions.We extract rules by fitting the training set to a DT model.

**Phase 2: hybrid model formation** In this phase, a hybrid model is formed by combining the rules extracted in each fold and the ML classifier, followed by the testing out of each model over the validation set using different parameters (e.g. lambda for logistic regression and gamma for SVM). For example, to form the first hybrid model, we combine the rule extracted from the first fold and a logistic regression model (i.e. rule + LR) and test out its performance over the validation set (left-out fold) while applying it to each of the 10 parameters. Thus, for each fold, we get 10 performance measures. We repeat this process for the succeeding folds, which means performing the steps for k times (i.e. with k = 9 as we are using 9-fold cross validation) will give us 90 performance measures. We compute the average performance across the folds. This will give us 10 average performance measures (i.e. for each parameter n) each corresponding to one specific hybrid model. Then we choose the best hybrid model (rule-based + regularized logistic regression), i.e. with lambda that gives a maximum average or the one that minimizes errors. We retrain using selected best hybrid model on all of D’ and test this out over the hidden test set T = ((*x*_1_,*y*_1_,…,(*x*_*t*_,*y*_*t*_)), i.e. 30 % of the dataset D and report the performance of the hybrid model. We evaluate the classifiers by their average performance for 20 repetitions of k-fold cross-validation using k = 9. We repeat the process for evaluation of other hybrid model (e.g. rule-based + SVM, rule-based + NB, etc.) and finally choose which hybrid model performs best.

**Fig. 13:**
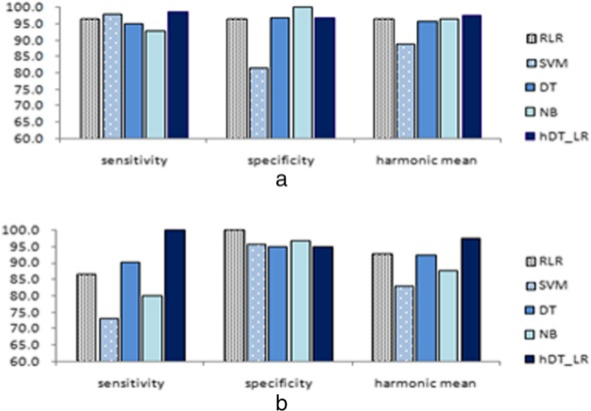
Comparison of the performance of RLR, SVM, DT, NB and Hybrid classifier hDT_RLR. **a** healthy and unhealthy classification (**b**) CP and PP type classification

The hybrid classifiers, SVMs, RLR, DT and NB were tested and our experiments demonstrate that hybrid classifier rule-based + RLR (hDT_RLR) achieves best performance for discriminating healthy from unhealthy (i.e. with paralysis) subjects. Similarly, for the palsy type classification: central palsy (CP) and peripheral palsy (PP), hDT_RLR hybrid classifier outperformed other classifiers used in the experiments. Figure 13 presents a graphical comparison of the average performance of our hybrid classifier, RLR, SVM, DT and NB based on our proposed approach. For healthy and unhealthy discrimination (Fig. 13a), our hDT_RLR hybrid classifier achieves a better performance on harmonic mean of almost 2.0 % higher than RLR, SVM, DT and NB. Similarly, for facial palsy classification, hDT_RLR is at least 4.2 % higher than the other classification methods as in Fig. 13b. Experiments reveal that hDT_RLR hybrid classifier yields more stable results.

**Fig. 14:**
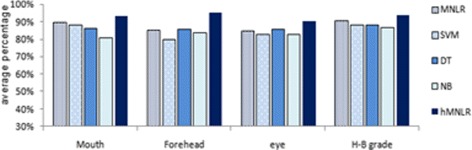
Comparison of the performance of RLR, SVM, DT, NB and Hybrid classifier hDT_RLR for region grading and overall H-B grade

For the quantitative assessment of the regional paralysis and overall FP grade, we consider Multinomial Logistic Regression (MNLR), SVM, Decision Tree (DT), naïve bayes (NB) and hybrid classifiers (e.g. rule-based + MNLR) as appropriate classification methods. Hybrid classifier hMNLR achieves best performance for facial palsy grading. Figure 14 presents a graphical comparison of the average performance of hMNLR, RLR, SVM, DT and NB based on the combined iris and key point-based approach. The accuracy of grading by hMNLR is at least 4 % higher than the average performance of the other five classification methods.

Tables 3 and 4 present the comparison of the classifier performance between the key point-based method using improved LAC model and the proposed approach combining iris and key point-based features. Overall, our proposed method outperforms the key point-based features in harmonic mean by at least 7.6 %; as well as the sensitivity and specificity with the improvement of 5.6 – 9.6 % for discriminating healthy from unhealthy subjects. Similarly, experiments show that our proposed approach yields better performance for classifying central and peripheral palsy particularly in sensitivity and specificity with an improvement of 5.7 – 6.2 %.

**Table 3:** Comparison of the performance of the two methods for healthy and unhealthy discrimination

	Key point-based	Our approach
Sensitivity	93.0 %	98.6 %
Specificity	87.1 %	96.7 %
Harmonic mean	90.0 %	97.6 %

**Table 4:** Comparison of the performance of the two methods for facial palsy classification

	Key point-based	Our approach
Sensitivity	93.6 %	99.3 %
Specificity	88.8 %	95.0 %
Harmonic mean	91.1 %	97.1 %

Some detailed comparisons of the performance of each of the classification methods including hybrid classifiers are presented in Tables 5 and 6.

**Table 5:** Comparison of the performance of different classifiers for facial palsy classification

Classifier	Sensitivity(%)	Specificity(%)	Harmonic mean(%)
RLR	86.7	100.0	92.9
SVM	73.3	95.8	83.1
DT	90.0	95.0	92.4
NB	80.0	96.7	87.5
hDT_RLR	99.3	95.0	97.1
hDT_SVM	99.3	90.8	94.9
hDT_NB	99.3	91.7	95.3
hLR_SVM	86.7	95.8	91.0
hLR_DT	93.3	94.5	93.9
hLR_NB	86.7	96.7	91.4
hSVM_LR	86.7	95.8	91.0
hSVM_DT	93.3	94.2	93.7
hSVM_NB	83.3	92.5	87.7
hNB_LR	86.7	96.7	91.4
hNB_DT	96.7	91.7	94.1
hNB_SVM	83.3	92.5	87.7

**Table 6:** Comparison of the performance of different classifiers for healthy and unhealthy discrimination

Classifier	Sensitivity(%)	Specificity(%)	Harmonic mean(%)
RLR	96.4	96.7	96.5
SVM	95.5	81.7	88.1
DT	95.0	96.7	95.8
NB	92.9	100.0	96.3
hDT_RLR	98.6	96.7	97.6
hDT_SVM	98.6	8.3	87.3
hDT_NB	95.0	6.7	95.8
hLR_SVM	95.6	78.3	86.1
hLR_DT	95.0	96.7	95.8
hLR_NB	95.0	96.7	95.8
hSVM_LR	95.6	96.7	96.1
hSVM_DT	92.9	96.7	94.7
hSVM_NB	92.9	100.0	96.3
hNB_LR	92.9	96.7	94.7
hNB_DT	92.9	96.7	94.7
hNB_SVM	98.6	78.3	87.3

Classification methods used in the experiments. Regularized Logistic Regression (RLR), Support Vector Machine (SVM), Decision Tree (DT), Naive Bayes (NB), combined DT+RLR (hDT_RLR), DT+SVM (hDT_SVM), DT+NB (hDT_NB), LR+SVM (hLR_SVM), LR+DT (hLR_DT), LR+NB (hLR_NB), SVM+LR (hSVM_LR), SVM+DT (hSVM_DT), SVM+NB (hSVM_NB), NB+LR (hNB_LR), NB+DT (hNB_DT), NB+SVM (hNB_SVM)

Hybrid classifiers noticeably reveal a significant improvement of the performance rather than using the classification methods individually. Although other hybrid classifiers also yield a good result as with the hDT_RLR, we need a hybrid classifier that provides stable results in terms of sensitivity performance measurement as it is more important in our study but of course without sacrificing the specificity; thus we chose hDT_RLR. A comparison of the performance of the proposed method based on iris-key-point-based feature extraction and the key-point-based approach for facial palsy grading is presented in Table 7. The proposed approach outperforms the key-point-based approach, most particularly in the regions of forehead and eye with the improvement of 6.0 and 5.2 % respectively. Most importantly an accuracy of almost 94 % for the overall H-B grading is achieved by our proposed iris-key-point-based approach.

**Table 7:** Comparison of the performance of the proposed method based iris segmentation and LAC model and the key point-based detection using LAC

	Key point-based	Our approach
Mouth	91.0 %	93.1 %
Forehead	89.0 %	95.0 %
eye	85.1 %	90.3 %
Overall H-B	90.1 %	93.7 %

The results demonstrate that the better performance is in the forehead and mouth region. Although eye region is noticeably having the lowest accuracy among other regions, but our proposed approach reveals a very significant improvement from using the key-point based approach. The approach based on the combined iris and LAC-based key point detection yields a better performance than the solely key point-based approach, most particularly in the forehead and eye region, which involves not only the key-point based features but as well as the iris behavior.

### Discussion

Empirical statistics and methods have found that active contour approach does have a very appealing quality that generates closed contours, which can be very useful in separating the outer boundaries of an object from the background than Canny and SUSAN [29, 31]. Therefore, it is presumed that localizing active contour (LAC) yields superior results than the standard edge detection tools used in the previous methods for detecting the landmark points of the human face. Good enough, our experiments show that LAC-based key point detection works well.

However, combining key point-based detection and iris segmentation for comprehensive facial paralysis assessment has yet more to offer as it outperformed the method that solely uses key points for feature extraction. Additionally, the eye region is full of wrinkles, especially the facial images of elderly people. The edges and eyelids ridge vary irregularly that sometimes deceived the system to generate asymmetrical results even for normal subjects. Features that are solely based on standard-edge-detection-tools-generated key points are not robust to model these subtle characteristics of eye surroundings.

Furthermore, our proposed approach to combine iris segmentation and the LAC-based key point detection for feature extraction provides a better discrimination of central and peripheral palsy most especially in ‘raising of eyebrows’ and ‘screwing of nose’ movements. It shows changes of the structure on edges of the eye, i.e., the significant difference between the normal side and the palsy side for some facial movements (e.g. eyebrow lifting, nose screwing, and showing of teeth). Also, features based on the combination of iris and key points generated by LAC can model the typical changes in the eye region. A closer look at the performance in the eye region, as shown in Tables 2, 3, 4 and 7 reveal interesting statistics in terms of the specific abilities of the two methods. Our method proves to have significant contribution in discriminating central from peripheral palsy patients and healthy from facial palsy subjects. The combination of iris segmentation and LAC-based key point approach is more suitable for this operation.

The system ‘fpQAS’ is implemented in matlab. The executable file is available for download on our website: http://infos.korea.ac.kr/fpQAS/.

## Conclusion

In this paper, we present a novel approach to quantitatively classify and assess facial paralysis in facial images. Iris segmentation and LAC-based key point detection are employed to extract the key features. The symmetry of facial images is measured by the ratio of the iris area and the vertical distances between key points in both sides of the face. Our Hybrid classifier provides an efficient quantitative assessment of the facial paralysis. One limitation of the proposed method is that it has a greater sensitivity to facial images, having significant natural bilateral asymmetry. However, our iris segmentation and key point-based method has several merits that are essential for our real application. Specifically, iris features describe the changes of the iris exposure while performing some facial expressions. They reveal the significant difference between the healthy side and the severe palsy side when raising eyebrows with both eyes directed upward, and can model the typical changes in the iris region.

Furthermore, iris-based features combined with key point-based features are insensitive to the illumination as our proposed method utilizes the key advantage of both localized active contour and the most cited algorithm for iris detection, the Daugman’s integro-differential operator.
